# Effects of Energy Intensification of Pressure-Swing Distillation on Energy Consumption and Controllability

**DOI:** 10.1021/acsomega.2c05959

**Published:** 2022-12-22

**Authors:** Jonathan Wavomba Mtogo, Andras Jozsef Toth, Daniel Fozer, Péter Mizsey, Agnes Szanyi

**Affiliations:** † Department of Chemical and Environmental Process Engineering, Budapest University of Technology and Economics, 1111Budapest, Hungary; ‡ Department of Fine Chemicals and Environmental Technology, University of Miskolc, 3515Miskolc, Hungary; § Chemical Engineering Division, Kenya Industrial Research and Development Institute, P.O. Box 30650, 00100Nairobi, Kenya; ∥ Department of Environmental and Resource Engineering, Technical University of Denmark, 2800Kgs. Lyngby, Denmark

## Abstract

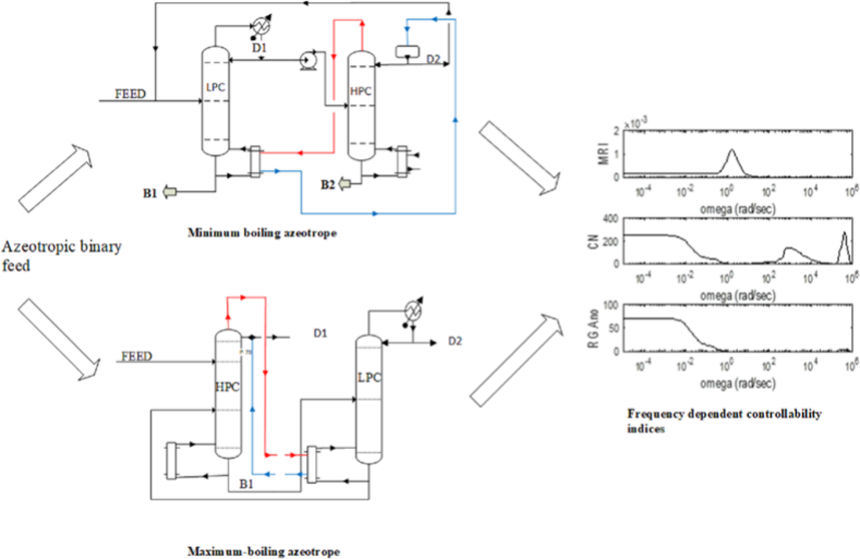

The aim of process integration is the efficient use of energy and natural resources. However, process integration can result in a more precise process operation, that is, it influences controllability. Pressure-swing distillation processes are designed for the separation of azeotropic mixtures, but their inherent heat integration option can be utilized to significantly reduce their energy consumption. One maximum-boiling and three minimum-boiling azeotropes are considered to study and compare the nonintegrated and integrated alternatives with the tool of mathematical modeling where ASPEN Plus and MATLAB software are used. The results show that the heat-integrated alternatives result in 32–45% energy savings that are proportional to the emission reduction and the consumption of natural resources. As far as the operability is concerned, the heat-integrated alternatives show worse controllability features than the nonintegrated base case. This can be due to the loss of one controllability degree of freedom. This recommends using more sophisticated control structures for the sake of safe operation if process integration is applied.

## 1 Introduction

Process integration is a powerful tool to improve process efficiencies. Such integration policy is also applied in the area of the separation of organic solvents. Organic solvents play a significant role in a variety of scientific and technical applications.^1^ Because of their efficiency in a wide range of unit operations, organic solvents have been used extensively in chemical processes. Industries using organic solvents create economic opportunities within their localities. However, the life cycles of industrial solvents introduce emission sources that may have an impact on the environment and human health.^2,3^ This may occur during the production, shipment, usage, and disposal of the solvents. In many circumstances, reclaiming and reusing solvents can reduce the life cycle impact of even the safest solvents.^2^ The technical challenge of solvent reprocessing is the application of appropriate separation technologies for solvent recovery and purification to desired quality specifications.

The nonideality behavior of vapor–liquid equilibrium in some binary mixtures results in the formation of minimum-boiling and maximum-boiling homogeneous azeotropes in some solvent systems.^4^ Minimum-boiling homogeneous azeotropes may form from dissimilar chemical components with strong molecular repulsion and activity coefficients greater than unity. Maximum-boiling homogeneous azeotropes may form if there is a molecular attraction between the chemical components with activity coefficients being less than unity.

When the composition of a binary homogeneous azeotrope changes considerably at different pressures, pressure-swing distillation (PSD) can effectively separate the binary components.^5−7^ Two distillation columns are employed in this process, each operating at a different pressure. The binary homogeneous azeotropes considered in this work are tetrahydrofuran–water, acetone–methanol, acetonitrile–water, and acetone–chloroform systems. The acetone–chloroform system constitutes a maximum-boiling azeotrope, and the others form minimum-boiling azeotropes. Tetrahydrofuran (THF), acetone, acetonitrile (ACN), methanol, and chloroform are solvents commonly used in chemical production.^8−10^ Industrial effluents containing these solvents must be treated so that there is no harm to the environment without the loss of resources.

Pressure-swing distillation has extensively been used for separating several azeotrope types, primarily binary minimum-boiling azeotropes^11−13^ and binary maximum-boiling azeotropes.^14−16^ In recent times, there has been a lot of interest in exploring the inherent heat integration option in PSD processes. Heat integration may happen in two ways: connecting the reboiler in the low-pressure column (LPC) to the condenser in the high-pressure column (HPC) or integrating the rectifying section in the HPC to the stripping section in the LPC.^12,17^ Both schemes provide significant energy-saving benefits. The heat-integrated pressure-swing distillation (HIPSD) process is an effective mode to save energy.^12,18^

The design and control of both nonheat-integrated PSD^19−21^ and HIPSD^12,14^ for separating azeotropes have been investigated. For example, Mtogo et al.^21^ worked on the THF–water system, designing a suitable control structure for a nonheat-integrated PSD using the desirability function. The identification of a tight control strategy, particularly for fully heat integration processes, is an integral step toward achieving safe and optimum operation for PSD systems because the neat configuration requirement is much stricter than that for ordinary PSD systems. Also, the control loop interactions are complex, making the controllability study a very important step.

The controllability of a process is its capability to run safely and profitably within constraints. This indicates the ability to achieve product purity design objectives in the presence of disturbances. As an inherent property of the process, controllability is considered at the preliminary design stage before fixing the control system design. Therefore, controllability has to be included as a design objective. A common approach in the evaluation of controllability is to perform dynamic simulations of the intended process. Since detailed dynamic simulations are time-consuming and require detailed system information, which may be unavailable at the early design stage, several authors^22−24^ have developed faster and simpler controllability study methods that are suitable for use in the early design phase. In the frequency domain, a key point to emphasize is that controllability analysis is applied for two cases: selection and pairing of the best controlled and manipulated variables within a process, and the ranking of control features for process alternatives. In our case, the PSD and HIPSD alternatives are used.

In our previous work,^21^ a comparison of the controllability features of extractive distillation and pressure-swing distillation for separating the minimum-boiling azeotrope of THF–water has been undertaken. Energy consumption for the two alternatives has been calculated and optimal controlled and manipulated variable pairings have been selected. The PSD was found to have better controllability. However, its energy consumption was higher.

Therefore, the purpose of this article is to investigate PSD and HIPSD for separating minimum-boiling and maximum-boiling binary homogeneous azeotropes based on energy consumption and controllability in the frequency domain and extend the methodology to heat-integrated systems with recycle flows.

So far, no research has been published that compares PSD with HIPSD for separating binary azeotropes through energy consumption and controllability angles in the frequency domain. Also, there has been limited research into the controllability studies of HIPSD for separating maximum-boiling azeotropes. This study is applied in the four chemical systems without and with full heat integration. Full heat integration is achieved by connecting the condenser of the high-pressure column to the reboiler of the low-pressure column. The effect of heat integration on energy consumption and process controllability is then analyzed. Only proportional-integral (PI) controllers are considered without using advanced process control strategies. Several control pairings are presented to provide stable regulatory-level plantwide control. The optimal variable pairings for controllability are deduced. The control design interface (CDI) component of Aspen Plus Dynamics is employed to achieve linearization. MATLAB is used to calculate controllability indices in the frequency domain, which are then aggregated into a single parameter using the desirability function. The individual controllability indices calculated by MATLAB are the Morari resilience index (MRI), conditioning number (CN), and relative gain array number (RGAno). The aggregate desirability is calculated using these indices. The PSD and HIPSD processes are ranked and evaluated using aggregate desirability. The optimal control strategy is chosen by comparing the desirability value of several control structures. This can provide some useful recommendations for industrial operation processes in the real world.

## 2 Steady-State Design

### 2.1 Pressure-Swing Distillation

In this work, one maximum-boiling azeotrope and two minimum-boiling azeotropes are adopted from existing publications to represent the PSD for binary azeotrope separation.^21,25−27^ For THF–water, acetone–water, and acetonitrile–water systems; binary feed flows, product purity levels, operating pressures of the columns, number of stages of the columns, and thermodynamic models are similar to the initial works. Sequential iterative optimization and heuristic optimization are methods commonly used in the optimization of PSD. Ghuge et al.^19^ used a heuristic optimization method to design a PSD process for separating THF–water and calculated the minimal TAC of the optimized system. The method was also used by Luyben^27^ for acetone–chloroform separation. These optimal values are selected in this work.

**Figure 1:**
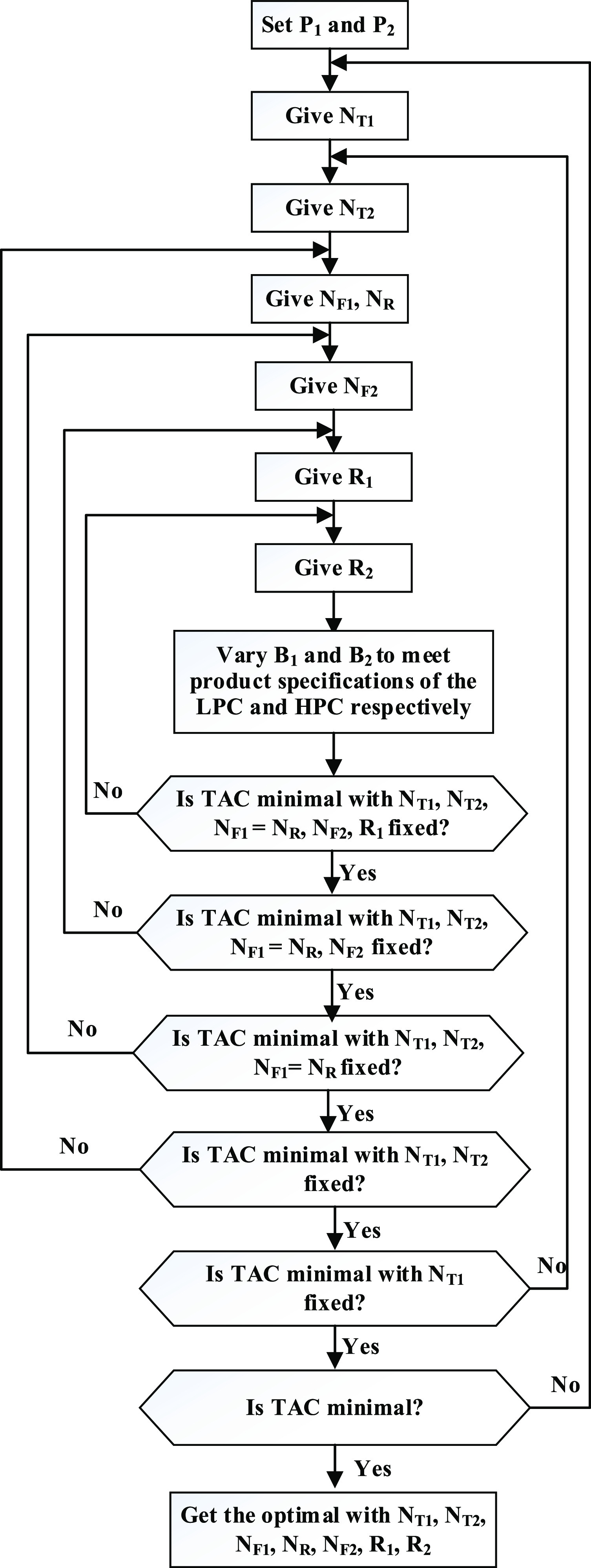
Optimization procedure for the acetone–methanol separation.

For the acetone–methanol system, a new simulation is made. The equimolar feed with a rate of 100 kmol/h at room temperature is fed into the LPC. The bottom stream of the LPC is 99.5% mole purity at 50 kmol/h. The overhead azeotrope is pumped into the HPC. The bottom product of the HPC is 99.5% mole purity acetone at 50 kmol/h, and the distillate stream of the HPC is the azeotrope that is recycled back. The selection of the operating pressures is shown in Section 2.1.1. The design variables include feed stages (N_F1_, N_R,_ and N_F2_), the molar reflux ratios (R1 and R2), and the total tray numbers (N_T1_ and N_T2_) of the LPC and HPC. The variables are optimized through the sequential iteration method. The objective function of the whole optimization process is to minimize the total annual cost (TAC). The optimization sequence is given in Figure 1.

For the other systems, the binary feed mixture is equimolar and has a 100 kmol/h flow rate at room temperature. The two columns operate at distinct pressures. High-purity product streams come out of one end of the column configuration. At the other end, recycle streams with compositions close to the two azeotropes are produced. For minimum-boiling azeotrope systems, the distillate of the second column is the recycle whereas, for the maximum-boiling azeotrope systems, the recycle stream is the bottoms from the second column. Rigorous Radfrac models are used to simulate the two columns in Aspen Plus.

Table 1 gives the thermodynamic property package used for each system. The default parameters for the chosen thermodynamic models are used. The product flows are fixed at 50 kmol/h, and in both columns, Aspen Design Spec/Vary is deployed to vary the reflux ratios and bring the compositions to their respective purity parameters. THF–water and acetonitrile–water have product purities of 99.9 mol %, while the other two systems have product purities of 99.5 mol %. In our previous work on controllability,^21^ we compared controllability at 95 and 99.9 mol % purity levels. We found that controllability was worse at higher purity levels. But for ranking, a selected control structure in the same distillation system for a particular separation will show the same rank compared to another. Therefore, purity levels of 99.5 and 99.9 mol % will not affect the ranking. The optimized flowsheet parameters are shown in Table 2.

**Table 1:** Thermodynamic Property Packages of the Mixtures

azeotropic system	thermodynamic property package
THF–water	NRTL
acetone–methanol	UNIQUAC
acetone–chloroform	UNIQUAC
acetonitrile–water	UNIQUAC

**Table 2:** Flowsheet Parameters for PSD

variables	THF–water	acetone–methanol	acetonitrile–water	acetone–chloroform
P_1_ (bar)	1	1	0.4	10
P_2_ (bar)	10	10	5	0.77
N_T1_/N_T2_	13/16	16/20	12/20	56/22
N_F1_/N_R_/N_F2_	10/10/8	8/8/10	9/9/10	14/34/17
R_1_/R_2_	0.22/0.29	2.15/0.29	0.30/0.36	27.64/26.10
ID_1_/ID_2_ (m)	0.83/0.63	2.18/1.06	1.05/0.76	2.46/2.24

**Figure 2:**
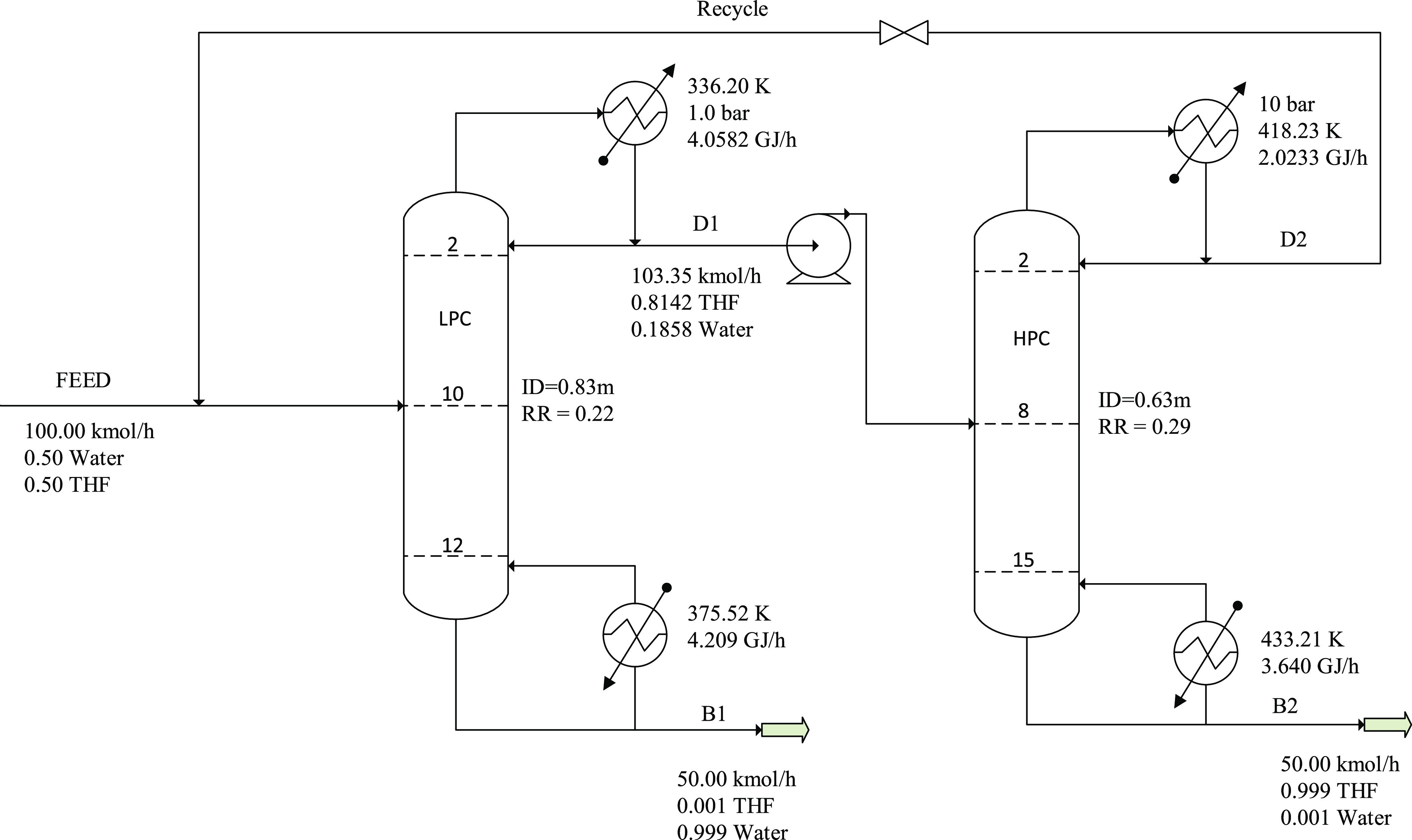
Optimal design process for the PSD without heat integration for THF dewatering.

**Figure 3:**
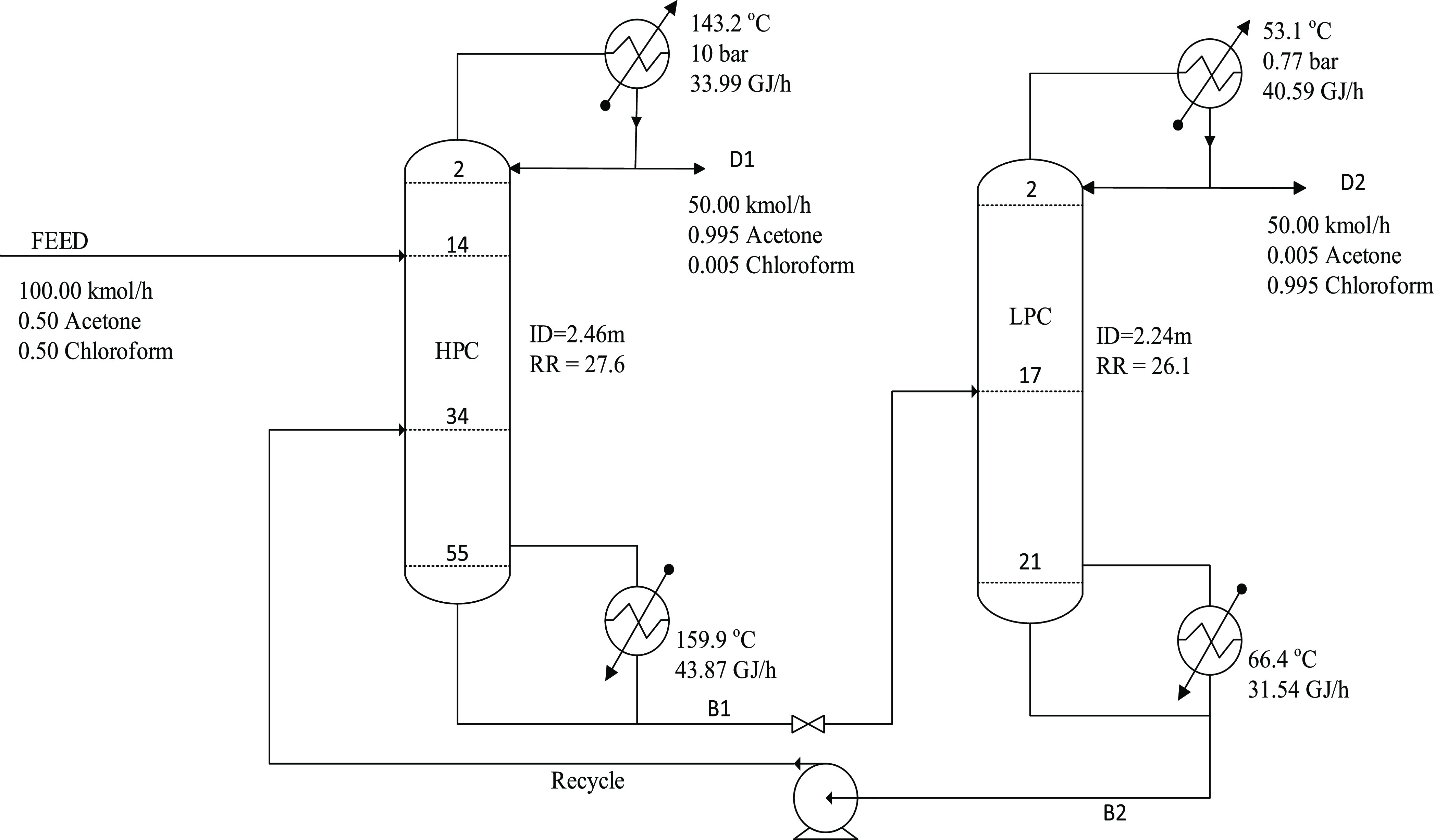
Optimal design process for the PSD without heat integration for the maximum-boiling azeotrope (acetone–chloroform).

Figure 2 depicts the flowsheet for THF–water separation with stream information, operating conditions, heat duties, and equipment sizes. This flowsheet scheme was used for all of the minimum-boiling azeotropes in this study. Figure 3 shows the flowsheet for the maximum-boiling azeotrope system.

#### 2.1.1 Selection of Pressures

The column pressures are selected on the basis of the pressure effect on azeotrope composition and reboiler temperatures. The operating pressure in the LPC is chosen to ensure the use of water as the coolant in the condenser. In the HPC, the operating pressure is set to allow for the use of high-pressure steam in the reboiler. The operating pressures, which are not optimized, are determined from the Txy curves of the azeotropic mixtures.

**Figure 4:**
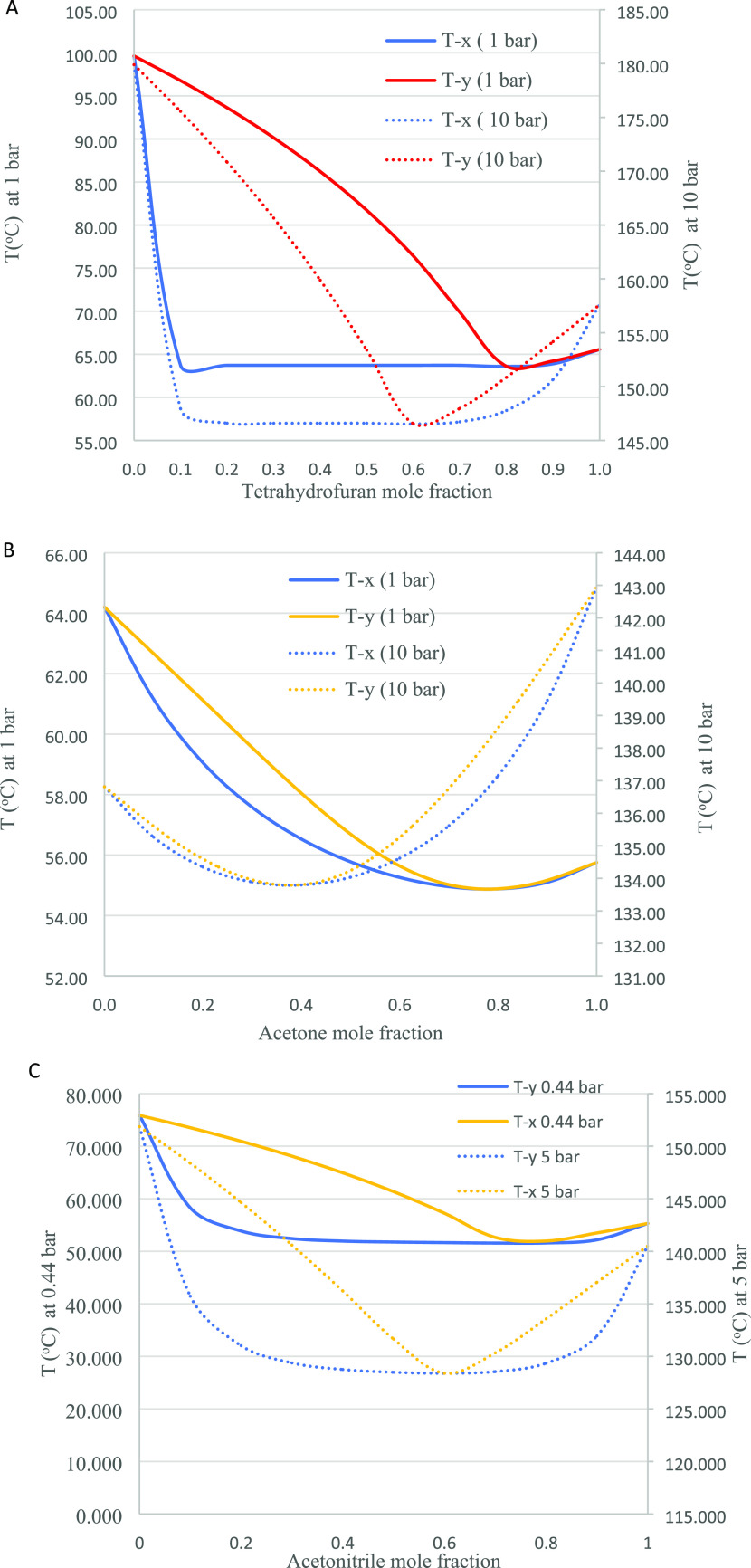

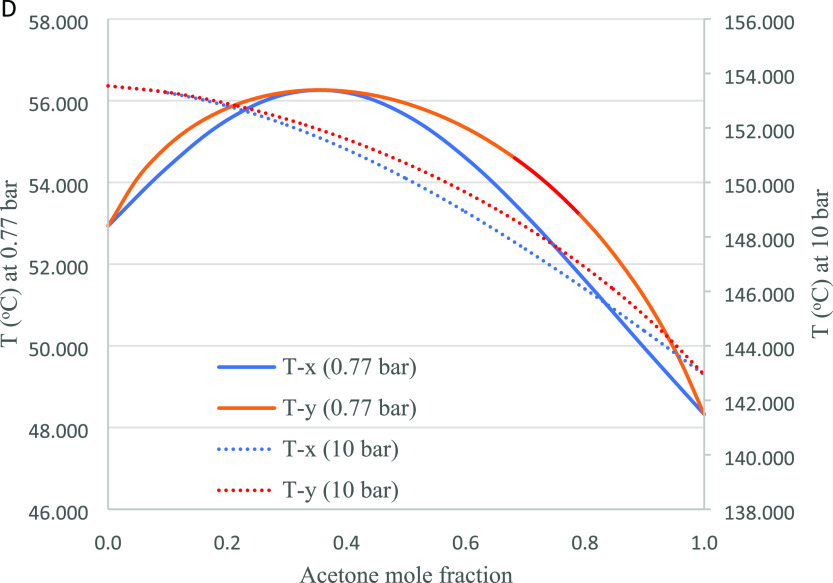
Txy diagrams for the binary azeotrope systems: (A) THF–water, (B) acetone–methanol, (C) acetonitrile–water, and (D) acetone–chloroform.

Figure 4 illustrates Txy diagrams for the systems at two different pressures, showing changes in the azeotropic composition in the four systems. The THF–water azeotrope at 1.0 bar has a composition of 82.90 mol % THF at 63.70 °C, while at 10.0 bar, the composition is 64.70 mol % THF at 147.90 °C. The acetone–methanol azeotrope at 1.0 bar has a composition of 78.30 mol % acetone at 55.00 °C, whereas at 10 bar, the composition is 31.50 mol % acetone at 134.80 °C. The acetonitrile–water azeotrope at 0.44 bar has a composition of 72.95 mol % acetonitrile at 53.75 °C, while at 5 bar, the composition is 60 mol % acetonitrile at 128.40 °C. The maximum-boiling acetone–chloroform azeotrope at 0.77 bar has a composition of 36.69 mol % acetone at 56.16 °C, while at 10 bar, the composition is 20.22 mol % acetone at 119.84 °C. All the azeotrope systems in this study have significant azeotropic composition shifts with pressure change.

### 2.2 Energy Intensification with Heat Integration

The feature that the two columns are at different pressures also makes their temperatures to be different. This gives the inherent opportunity to complete heat integration in pressure-swing distillation. The condenser of the high-pressure column can be matched with the reboiler of the low-pressure column (Figure 4). Such heat integration enables HPC to heat up LPC. Table 3 gives temperature differences. There is a sufficient temperature difference to provide a sufficient heat transfer area in each system. The overall heat transfer coefficient of 0.00306 GJ h^–1^ m^–2^ °C^–1^ is adapted from similar works.^27−29^ The heat loads of the reboilers are considered, since these are the most cost-effective parts of the energy consumption.

**Table 3:** Temperature Differences and Heat Transfer Areas

binary azeotropic system	reflux drum temperature in HPC (°C)	base temperature in LPC (°C)	temperature differences (°C)	heat transfer area (m^2^)
THF–water	145.08	102.38	42.70	32.23
acetone–methanol	134.93	66.54	68.39	74.56
acetone–chloroform	143.21	66.37	76.84	136.1
acetonitrile–water	129.88	80.10	49.78	40.924

**Figure 5:**
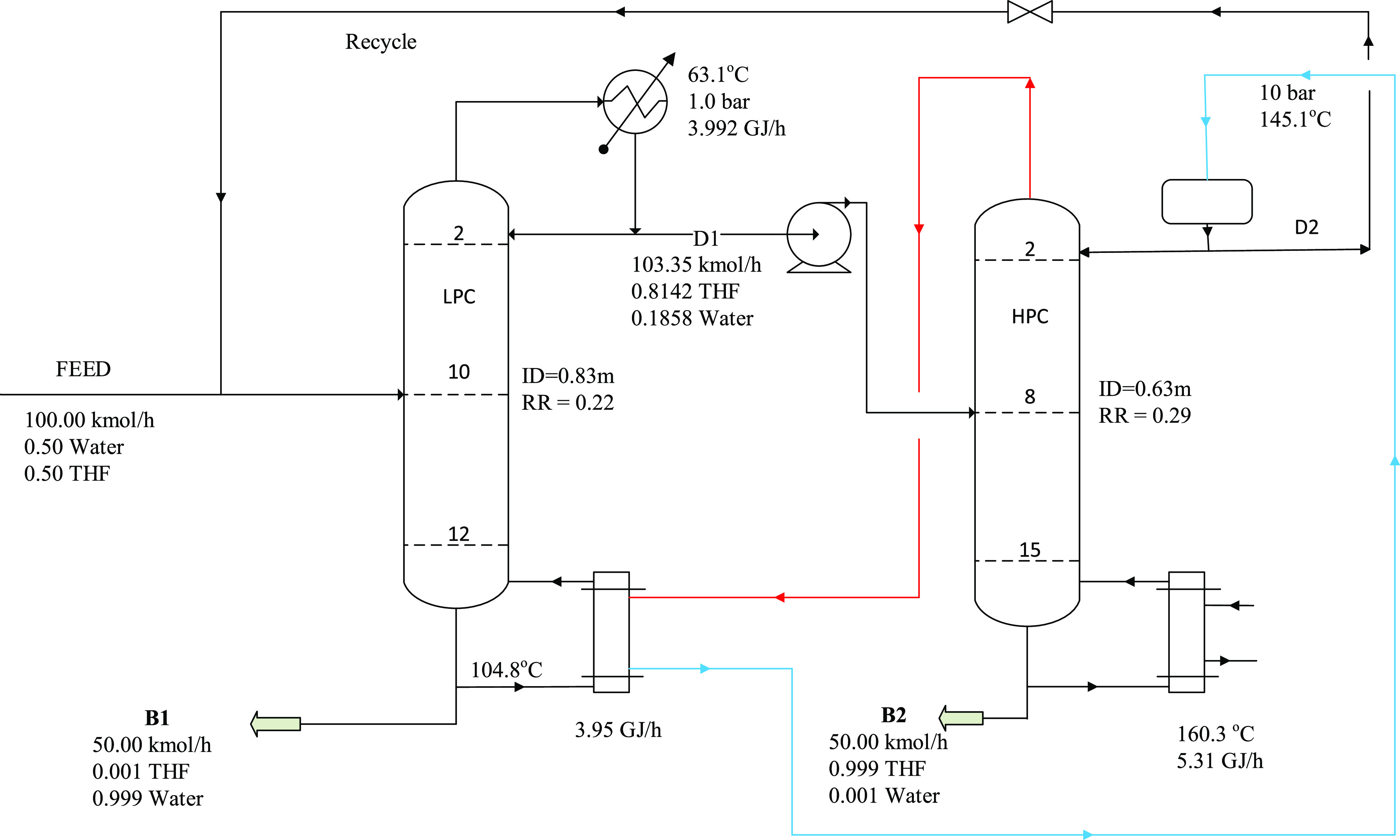
Optimal design process for the HIPSD for THF dewatering.

**Figure 6:**
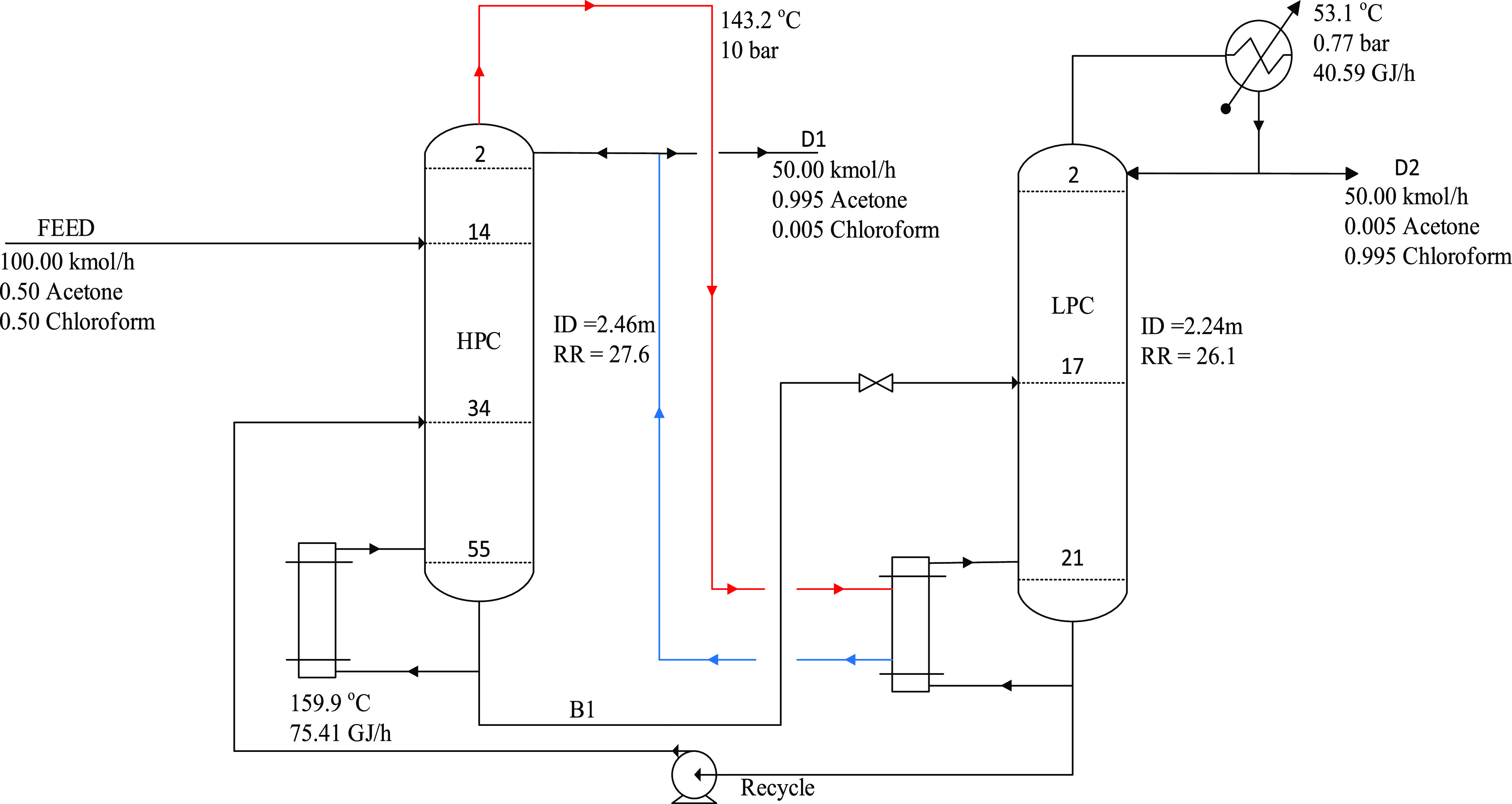
Optimal design process for the HIPSD for the maximum-boiling azeotrope (acetone–chloroform).

The reflux ratio is set in the first column, and then three variables (product flows and R2) are manipulated to achieve the appropriate purity levels for the two product compositions and equate the heat duties. Figures 5 and 6 give the heat-integrated flowsheet.

The energy requirements for all azeotropic systems and the separation alternatives are shown in Table 4. It can be seen that heat savings are achievable through heat integration.

**Table 4:** Comparison of Energy Consumptions for PSD and HIPSD

	energy consumptions, GJ/h		
	PSD	HIPSD	energy reduction, %
THF–water	7.85	5.31	32.36
ACN–water	11.83	7.45	37.02
ACE–MET	31.95	18.10	43.35
ACE–CHLR	75.41	41.40	45.10

The energy saving achieved by full heat integration is maximum in the acetone–methanol system. The separation of the maximum-boiling azeotrope of acetone–chloroform is energy-intensive in comparison with the other systems. This is because of the less sensitivity of the azeotrope to pressure changes.

## 3 Dynamic Control Analysis

**Figure 7:**
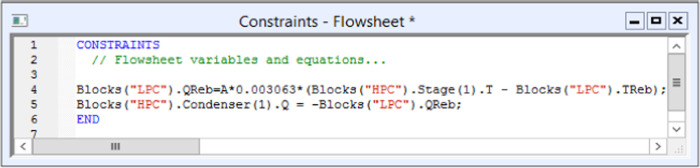
Aspen Plus Dynamics equations for heat integration.

The first and last stage liquid flows are used to calculate the reflux drum sizes and the column base sizes. These are sized to give 10 min of liquid holdup when full.^30^ The flowsheet equations are employed in the dynamic simulation to accomplish complete heat integration. Figure 7 shows the heat integration calculation formula, where A stands for the heat transfer area.

The heat duty of the LPC reboiler is calculated in the first row, and the duty of the HPC condenser is calculated in the second row.

The heuristic criterion on the control structure design of selecting the closest probable manipulated variable is used to choose variables for manipulating product compositions. The product A composition is determined by the first manipulated variable per pairing, whereas the product B composition is determined by the second. These pairings are shown in Table 5. PI controllers are used, which are tuned by the Ziegler–Nichols methods, and time constants are calculated through load rejection examinations in feed flow and composition disturbances.

**Figure 8:**
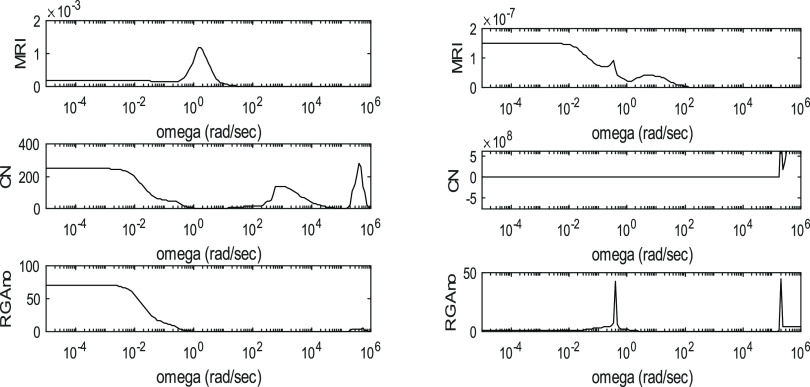
Controllability indices of PSD and HIPSD in the case of the THF–water system for the R1–R2 manipulated variable sets.

**Table 5:** Pairing of Control and Manipulated Variables

separation system	minimum-boiling PSD	maximum-boiling PSD	minimum-boiling HIPSD	maximum-boiling HIPSD
controlled compositions	X_B1_–X_B2_	X_D1_–X_D2_	X_B1_–X_B2_	X_D1_–X_D2_
set 1 of manipulated variables	R1–R2	R1–R2	R1–R2	R1–R2
set 2 of manipulated variables	Q1–Q2	Q1–Q2	R1–Q2	Q1–R2

Control investigations of both the PSD and HIPSD systems are carried out through the approach introduced by Gabor and Mizsey^24^ of the calculation of controllability indices in the frequency domain. This fastest technique uses the control design interface (CDI) of Aspen Plus Dynamics for linearization and calculates state-space matrices for each pairing of input and output variables. The controllability indices can then be obtained from the calculations of state-space matrices. A script in Aspen is created containing the input and output variables, as well as the relevant functions of the system under investigation to calculate the various matrices of the state-space model. It is essential that the input variables are always fixed and the output variables are always free in the simulation. After attaining the steady state, the simulation must be terminated and the script must be executed. Output files are generated, each of which contains basic information about the results as well as the various matrices of the state-space model in the sparse matrix form. From the transmitted matrices, a MATLAB code is written to present the controllability indices as graphs in the frequency domain. The presented controllability indices are the Morari resiliency index (MRI), condition number (CN), and relative gain array number (RGAno).

These indices are described for the open-loop frequency function matrix. MRI is the least singular value of this matrix.^31^ CN is the ratio between the largest and smallest singular values of this matrix. Better controllability is indicated by large values of MRI. CN values between 1 and 10 are generally acceptable. Values of CN higher than 100 indicate a less controllable process. RGAno is described as the sum of the absolute values of the relative gain array elements minus the identity matrix.
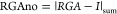
where *I* represents the identity matrix and RGA is the relative gain array, which is defined as follows
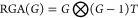
where *G* is a nonsingular square matrix and  depicts the multiplication of element by element. The transpose of the corresponding matrix is indicated by *T*.

For identifying optimal pairings that correspond to good controller performance, the relative gain array (RGA) is applied. It refers to how the control loops in the process interact with one another. Pairings with weaker interactions, as indicated by low RGAno, are the ones desired. The three controllability indices are computed over a frequency range that provides an insight into the dynamic controllability features of each system. For illustration, Figure 8 shows the controllability indices for the THF–water separation. The figures for the other three azeotropic systems and control variable pairings are provided in Supporting Information.

For each of the three controllability indices, a desirability function^32,33^ is calculated. The geometric mean of the individual desirability functions gives rise to the aggregated desirability function for a particular system.

The calculations for the desirability functions are achieved using the following formulae
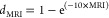

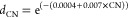

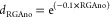

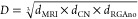
where *D* is the aggregated desirability function for a particular system.

This aggregated result provides the means to directly compare different controllability alternatives. Good process controllability is indicated by *D* values closer to 1.

## 4 Results and Discussion

The calculations of the controllability studies of the azeotropic systems are shown in Table 6 for PSD and in Table 7 for HIPSD. Step changes in the setpoint value of closed-loop simulations are used to obtain time constants. The frequency was then calculated from the time constant.

**Table 6:** Controllability Indices and Desirability Values for the PSD Mode

	THF–water		ACN–water		acetone–methanol		acetone–chloroform	
control structure	R1–R2	Q1–Q2	R1–R2	Q1–Q2	R1–R2	Q1–Q2	R1–R2	Q1–Q2
time constant (h)	0.3	0.3	0.4	0.4	0.55	0.55	0.6	0.6
frequency (rad/s)	9.26 × 10^–4^	9.26 × 10^–4^	6.94 × 10^–4^	6.94 × 10^–4^	5.05 × 10^–4^	5.05 × 10^–4^	4.63 × 10^–4^	4.63 × 10^–4^
MRI	1.72 × 10^–4^	5.01 × 10^–3^	1.61 × 10^–5^	4.81 × 10^–5^	7.92 × 10^–3^	1.29 × 10^–2^	1.54 × 10^–2^	1.35 × 10^–3^
CN	2.47 × 10^2^	5.01 × 10^1^	1.24 × 10^2^	2.40 × 10^1^	1.60 × 10^2^	1.47 × 10^2^	2.52 × 10^1^	4.00 × 10^2^
RGAno	7.10 × 10^1^	1.59 × 10^1^	4.98 × 10^1^	7.70 × 10^0^	4.13 × 10^1^	3.97 × 10^1^	7.66 × 10^0^	3.74 × 10^2^
dMRI	1.72 × 10^–3^	4.89 × 10^–2^	1.61 × 10^–4^	4.80 × 10^–4^	7.62 × 10^–2^	1.21 × 10^–1^	1.43 × 10^–1^	1.34 × 10^–2^
dCN	1.78 × 10^–1^	7.04 × 10^–1^	4.21 × 10^–1^	8.45 × 10^–1^	3.26 × 10^–1^	3.56 × 10^–1^	8.38 × 10^–1^	6.10 × 10^–2^
dRGAno	8.26 × 10^–4^	2.05 × 10^–1^	6.88 × 10^–3^	4.63 × 10^–1^	1.61 × 10^–2^	1.89 × 10^–2^	4.65 × 10^–1^	6.01 × 10^–17^
aggregate desirability	6.32 × 10^–3^	1.92 × 10^–1^	7.75 × 10^–3^	5.73 × 10^–2^	7.37 × 10^–2^	9.33 × 10^–2^	3.82 × 10^–1^	3.66 × 10^–7^

**Table 7:** Controllability Indices and Desirability Values for the HIPSD Mode

	THF–water		ACN–water		acetone–methanol		acetone–chloroform	
control structure	R1–R2	R1–Q2	R1–R2	R1–Q2	R1–R2	R1–Q2	R1–R2	Q1–R2
time constant (h)	1	1	1.134	1.134	2	2	0.68	0.68
frequency (rad/s)	2.78 × 10^–4^	2.78 × 10^–4^	2.45 × 10^–4^	2.45 × 10^–4^	1.39 × 10^–4^	1.39 × 10^–4^	4.06 × 10^–4^	4.06 × 10^–4^
MRI	1.39 × 10^–7^	6.50 × 10^–8^	8.56 × 10^–6^	2.29 × 10^–5^	1.15 × 10^–3^	6.09 × 10^–3^	2.40 × 10^–3^	1.56 × 10^–3^
CN	2.72 × 10^3^	3.29 × 10^1^	4.59 × 10^2^	3.18 × 10^2^	6.32 × 10^2^	7.37 × 10^1^	2.91 × 10^2^	5.11 × 10^2^
RGAno	5.35 × 10^–1^	6.93 × 10^–1^	2.24 × 10^2^	1.11 × 10^2^	5.16 × 10^1^	4.07 × 10^1^	8.26 × 10^1^	1.79 × 10^2^
dMRI	1.39 × 10^–6^	6.50 × 10^–7^	8.56 × 10^–5^	2.29 × 10^–4^	1.15 × 10^–2^	5.91 × 10^–2^	2.37 × 10^–2^	1.55 × 10^–2^
dCN	5.46 × 10^–9^	7.94 × 10^–1^	4.03 × 10^–2^	1.08 × 10^–1^	1.20 × 10^–2^	5.97 × 10^–1^	1.30 × 10^–1^	2.79 × 10^–2^
dRGAno	9.48 × 10^–1^	9.33 × 10^–1^	1.80 × 10^–10^	1.50 × 10^–5^	5.74 × 10^–3^	1.72 × 10^–2^	2.59 × 10^–4^	1.70 × 10^–8^
aggregate desirability	1.93 × 10^–5^	7.84 × 10^–3^	8.53 × 10^–6^	7.19 × 10^–4^	9.25 × 10^–3^	8.46 × 10^–2^	9.29 × 10^–3^	1.94 × 10^–4^

MRI and CN require open-loop transfer function matrices. These are generated by changes in the manipulated variables and the dynamic responses of the products. For each distillation system presented in this work, two controlled variables that represent product purities were considered. Also, two manipulated variables were selected for each system. Generally, higher values of MRI and lower values of CN are preferred. It can be seen that the MRI values for all of the PSD systems are low. For the minimum-boiling azeotropes, the Q1–Q2 structure has relatively higher MRI values and lower CN values than R1–R2. Both indices indicate better control properties for the Q1–Q2 structure. The MRI and CN indices showed similar comparison results. For the maximum-boiling azeotrope, Q1–Q2 has a lower MRI value and higher CN values than R1–R2. This indicates poorer control properties for Q1–Q2. However, appropriate pairings are shown by the RGAno. The RGAno takes the control loop interactions into consideration. The weaker the interactions, the more desirable the pairing. Therefore, very low RGAno values are desired. For the minimum-boiling systems, Q1–Q2 has the lower RGAno. For the maximum-boiling acetone–chloroform, R1–R2 has the lowest RGAno.

In the case of PSD, better process controllability for the minimum-boiling azeotrope is achieved by the Q1–Q2 control configuration. For the maximum-boiling azeotrope, R1–R2 gives better controllability. This is because the products are distillates and the reflux ratios are closer to the manipulated variables.

For HIPSD, the control configuration where manipulated variables are close to the control variables also gives better controllability. However, for azeotropic separation of the same binary system, HIPSD shows poor controllability than PSD. This is due to the loss of the control degree of freedom created by the inability to separately set the heat input to the reboiler of the LPC. Instead, it is equivalent to the rate of heat removal in the condenser of the HPC. The operating pressure of the HPC cannot be controlled in HIPSD because the heat released by condensing overhead vapor of the HPC is totally used to provide the reboiler heat duty of the LPC. Therefore, only small disturbances can be handled by HIPSD.

## 5 Conclusions

Energy intensification is a crucial part of the chemical process design. A promising alternative to accomplish these goals is pressure-swing distillation, since it has an inherent heat integration option due to the two columns operating at different pressures. This separation alternative is studied in the case studies of three minimum-boiling azeotropes and one maximum-boiling azeotrope. The simultaneous study of the effect of heat integration on energy consumption and controllability in the case of the separation of azeotropic systems proves to be an interesting new direction in chemical process design.

Significant energy reduction is found (32–45%). The emission reduction and the saving of natural resources can be considered proportional to these numbers. However, heat integration influences controllability features. In contrast to previous studies, dynamic controllability indices are determined in the frequency domain and compared for the four nonintegrated and four integrated pressure-swing distillation alternatives. In agreement with common sense, it is proved that heat integration makes the controllability features worse, since one degree of freedom is lost due to the match of the two columns.

These results prove the importance of energy intensification carried out with process integration, but this is associated with more severe householding also including the controllability and the design of a more sophisticated control structure.

## Supporting Information Available

Controllability indices in the case of the THF–water system for the Q1–Q2 and R1–Q2 manipulated variable sets for PSD and FHIPSD, respectively; controllability indices of PSD and FHIPSD in the case of the acetone–chloroform system for the R1–R2 manipulated variable sets; controllability indices in the case of the acetone–chloroform system for the Q1–Q2 and Q1–R2 manipulated variable sets for PSD and FHIPSD, respectively; controllability indices of PSD and FHIPSD in the case of the acetone–methanol system for the R1–R2 manipulated variable sets; controllability indices in the case of the acetone–methanol system for the Q1–Q2 and R1–Q2 manipulated variable sets for PSD and FHIPSD, respectively; controllability indices of PSD and FHIPSD in the case of the acetonitrile–water system for the R1–R2 manipulated variable sets; and controllability indices in the case of the acetonitrile–water system for the Q1–Q2 and R1–Q2 manipulated variable sets for PSD and FHIPSD, respectively (PDF)

## Nomenclature

ACE: acetone
ACN: acetonitrile
CDI: control design interface
CHLR: chloroform
CN: conditioning number
HIPSD: heat-integrated pressure-swing distillation
HPC: high-pressure column
ID: diameter of the column, m
LPC: low-pressure column
MET: methanol
MRI: Morari resiliency index
PSD: pressure-swing distillation
Q1: reboiler heat load of the first column
Q2: reboiler heat load of the second column
R1: reflux ratio of the first column
R2: reflux ratio of the second column
RGAno: relative gain array number
THF: tetrahydrofuran
*x*: product composition
