# Supplementary material for: Effects of Energy Intensification of Pressure-Swing Distillation on Energy Consumption and Controllability
Source: ACS Omega. 2022 Dec 22;8(1):726–36. doi: 10.1021/acsomega.2c05959 (PMC9835167; doi:10.1021/acsomega.2c05959)
Supplement: Supplementary file 1 — ao2c05959_si_001.pdf [file ao2c05959_si_001.pdf]

Effects of energy intensification of pressure swing distillation on energy consumption and controllability

Jonathan Wavomba Mtogo[a,c], Andras Jozsef Toth[a], Daniel Fozer[d], Peter Mizsey[a,b,*], Agnes Szanyi[a]

[a]*Department of Chemical and Environmental Process Engineering, Budapest University of Technology and Economics, 1111 Budapest, Hungary*

[b]*Department of Fine Chemicals and Environmental Technology, University of Miskolc, 3515 Miskolc, Hungary*

[c]*Chemical Engineering Division, Kenya Industrial Research and Development Institute, P.O. Box 30650 – 00100 Nairobi, Kenya*

[d]*Department of Environmental and Resource Engineering, Technical University of Denmark, 2800 Kgs. Lyngby, Denmark*

[*]Corresponding author: mizsey@mail.bme.hu

Supporting Information

1. Controllability indices

The controllability indices of PSD and FHIPSD for the four azeotropic systems are shown from Figure S1 -Figure S7.

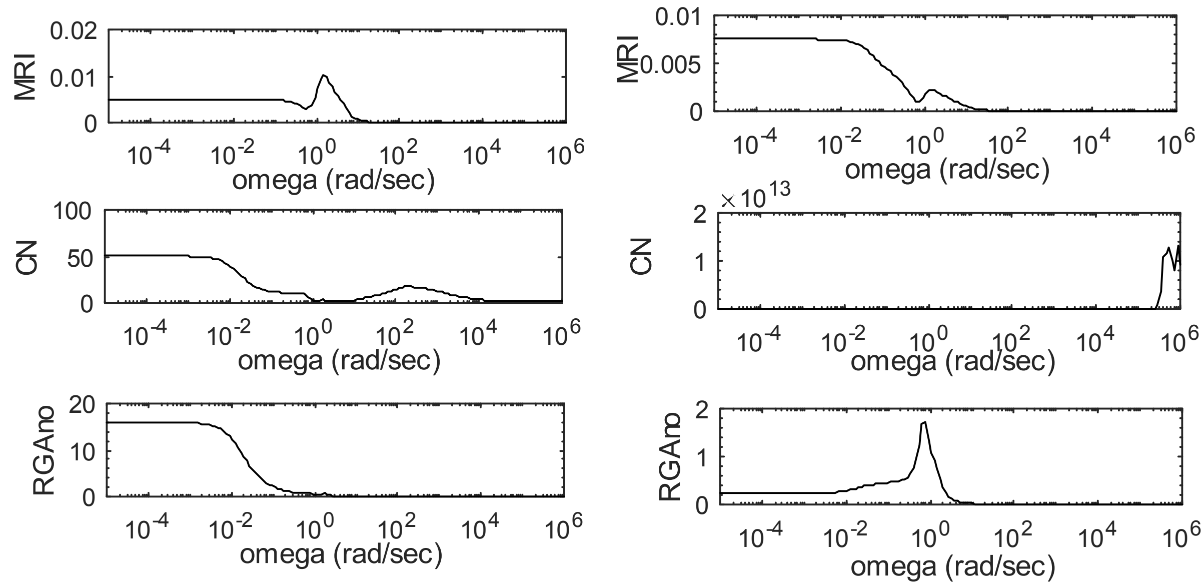

Figure S1:Controllability indices in the case of THF/water system for the Q1-Q2 and R1-Q2 manipulated variable sets for PSD and FHIPSD respectively.

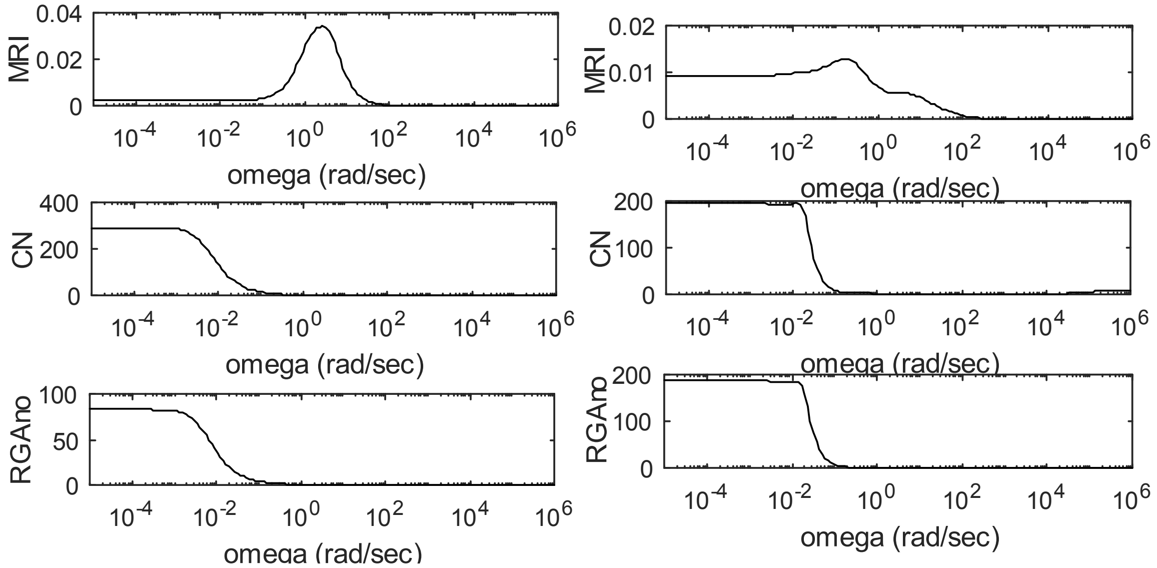

Figure S2:Controllability indices of PSD and FHIPSD in the case of acetone/chloroform system for the R1-R2 manipulated variable sets

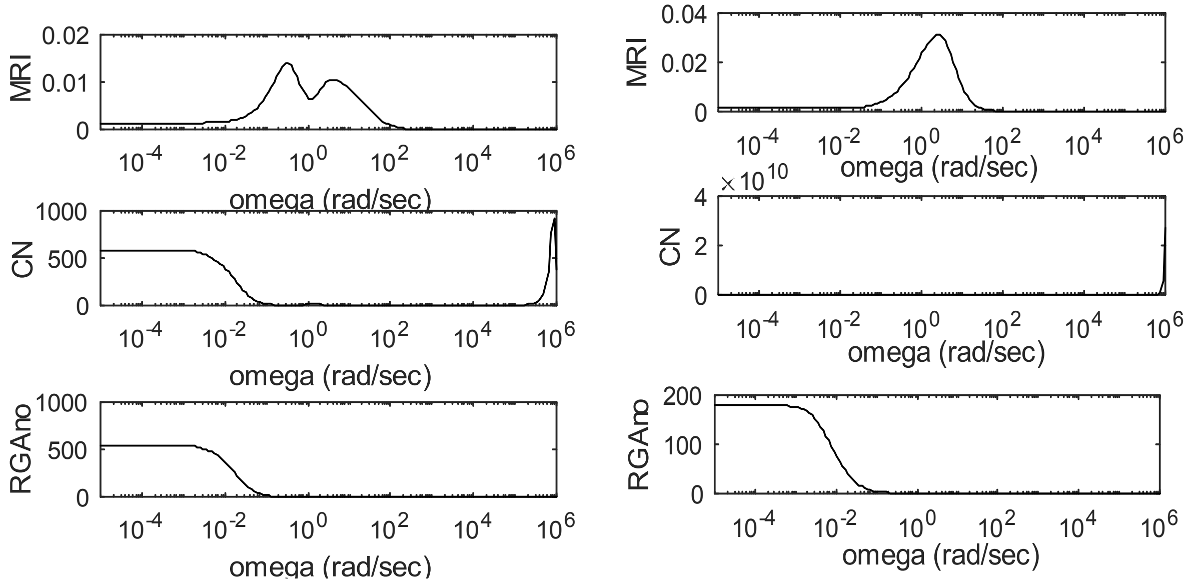

Figure S3:Controllability indices in the case of acetone/chloroform system for the Q1-Q2 and Q1-R2 manipulated variable sets for PSD and FHIPSD respectively.

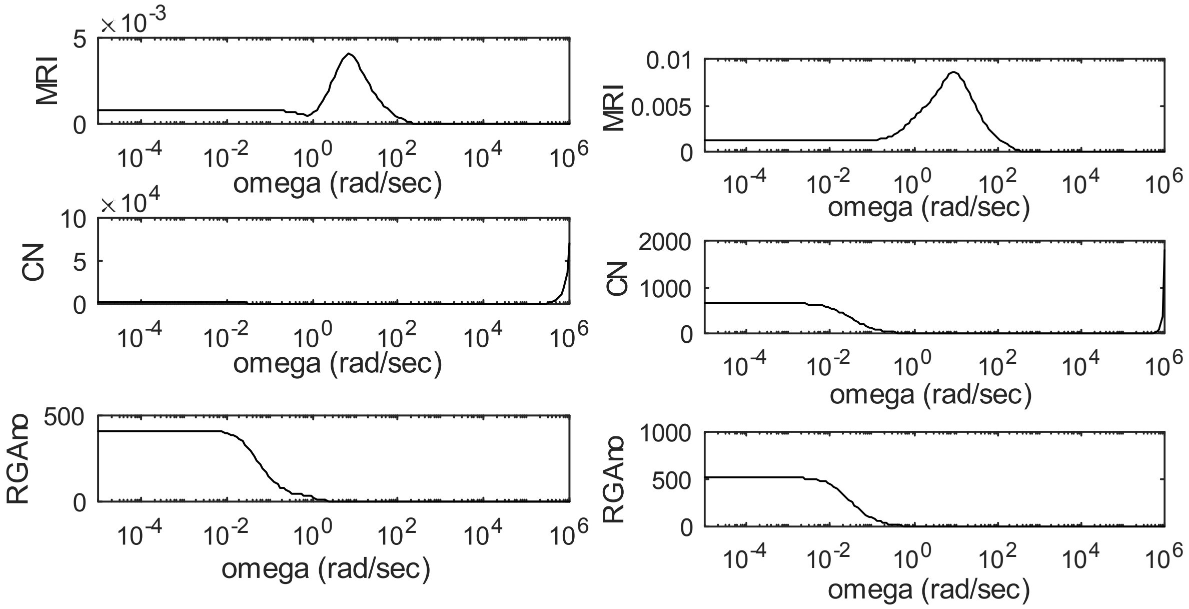

Figure S4:Controllability indices of PSD and FHIPSD in the case of acetone/methanol system for the R1-R2 manipulated variable sets.

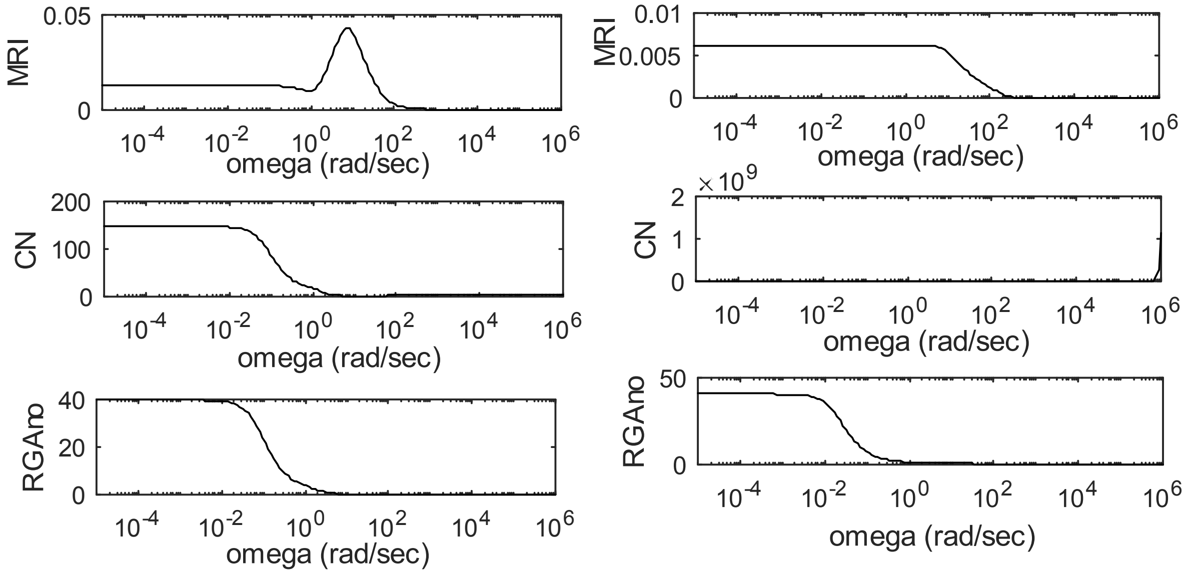

Figure S5:Controllability indices in the case of acetone/methanol system for the Q1-Q2 and R1-Q2 manipulated variable sets for PSD and FHIPSD respectively.

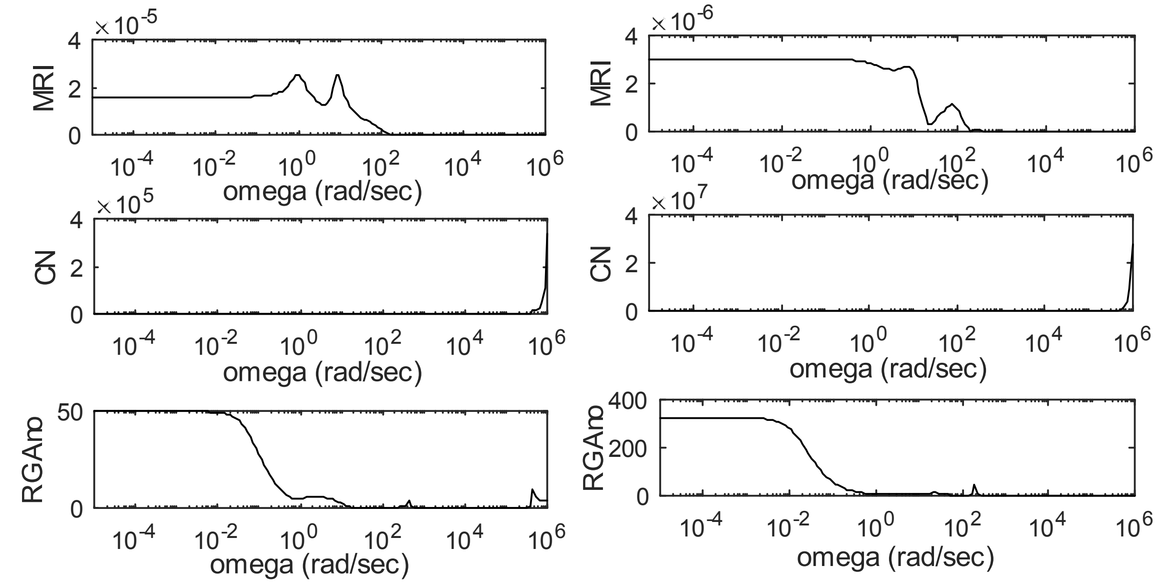

Figure S6:Controllability indices of PSD and FHIPSD in the case of acetonitrile/water system for the R1-R2 manipulated variable sets.

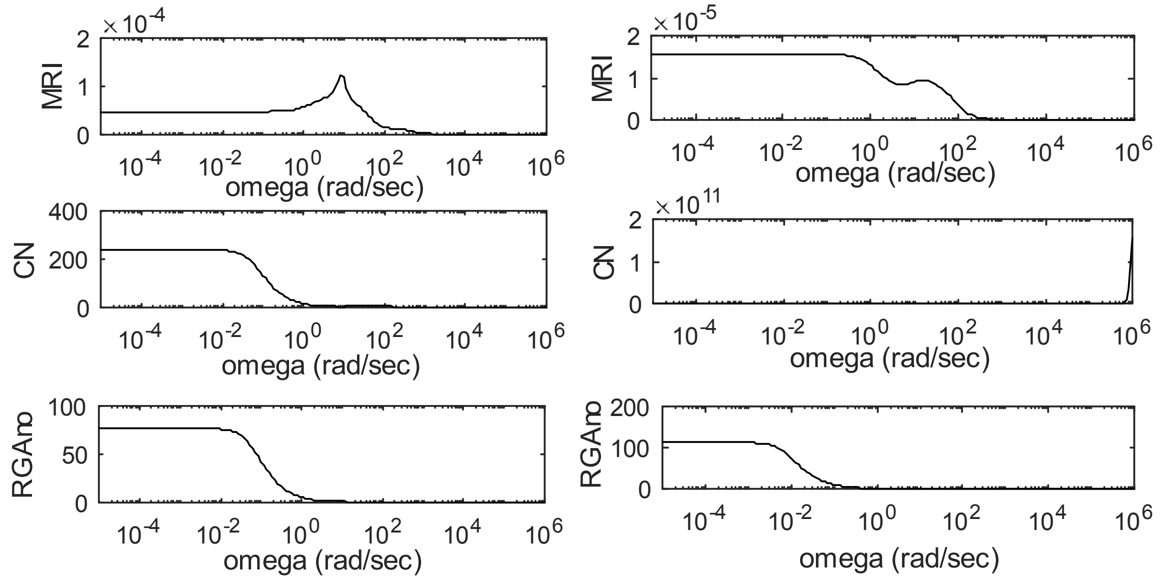

Figure S7:Controllability indices in the case of acetonitrile/water system for the Q1-Q2 and R1-Q2 manipulated variable sets for PSD and FHIPSD respectively.
